# Supplementary material for: How Long Is the Brain Perfusable After Global Ischemia? A Systematic Review
Source: Brain Sci. 2026 Aug 19;16(8):882. doi: 10.3390/brainsci16080882 (PMC13510562; doi:10.3390/brainsci16080882)
Supplement: Supplementary file 1 [file brainsci-16-00882-s001.zip › DataS7_Spatial-completeness-of-perfusion.html]

Spatial completeness of perfusion


# Spatial completeness of perfusion

**Perfusate:** 
● Pure blood 
● Carbon black 
● Mixed blood 
● Other non-blood

**Shape:** 
● n reported 
▲ n not reported

**Size:** 
larger points = larger n per arm

Hover over points for study details. Click a point to open the source paper.
