# Supplementary material for: How Long Is the Brain Perfusable After Global Ischemia? A Systematic Review
Source: Brain Sci. 2026 Aug 19;16(8):882. doi: 10.3390/brainsci16080882 (PMC13510562; doi:10.3390/brainsci16080882)
Supplement: Supplementary file 1 [file brainsci-16-00882-s001.zip › DataS8_Frequency-of-adequate-perfusion.html]

Frequency of adequate perfusion


# Frequency of adequate perfusion

**Perfusate:** 
● Pure blood 
● Carbon black 
● Mixed blood 
● Other non-blood

**Shape:** 
● Review-defined adequacy criterion 
▲ Study-defined adequacy criterion

**Size:** 
larger points = larger n per arm; unreported n plotted at minimum size

**Trend:** 
LOESS smoothers are shown for pure blood and carbon black as descriptive trends; no inferential confidence intervals are shown.

Hover over points for study details. Click a point to open the source paper.
